# The Effect of an 8-Week Vegan Diet on the Nutritional Status and Performance of Semi-Professional Soccer Players—Results of the VegInSoc Study

**DOI:** 10.3390/nu17142351

**Published:** 2025-07-17

**Authors:** Josefine Nebl, Pauline Bruns, Meike Meier, Frank Mayer, Martin Smollich, Markus Keller

**Affiliations:** 1 Research Institute for Plant-Based Nutrition, 35444 Biebertal, Germany; 2 Institute of Nutritional Medicine, University Hospital of Schleswig-Holstein, 23538 Lübeck, Germany; 3 University Outpatient Clinic, Center of Sports Medicine, 14469 Potsdam, Germany

**Keywords:** veganism, sports nutrition, soccer, nutritional status

## Abstract

Background/Objectives: Although there is an increasing interest among athletes in adopting plant-based diets, there is insufficient research available to determine how a vegan diet affects soccer performance. Methods: This interventional pilot study examined the effect of an 8-week vegan diet (VEG, n = 10) on nutritional status and athletic performance in semi-professional soccer players compared to controls (CON, n = 8). The study employed a controlled, non-randomized, longitudinal pilot study design during the season to compare the two groups. Results: Both groups displayed overall differences in nutrient intake, including insufficient energy and carbohydrates (t2: 46.2 [40.3–52.2] En% (VEG) vs. 37.6 [34.1–41.1] En% (CON); *p* = 0.036, Cohen’s *d* = 1.321). Notably, biochemical parameters 25(OH)D and ferritin levels fell within the normal ranges for both groups. The VEG group exhibited favorable changes in total and LDL cholesterol levels. Both groups had increased performances on the treadmill over the entire course of the study (VEG: +0.87 km/h (6.6%); CON: +0.96 km/h (7%); *p* > 0.05). The initial relative VO_2max_ at t0 was comparable between the groups. Primarily due to the significant weight loss in the VEG group (−1.94 kg, *p* = 0.007) rather than a change in absolute VO_2max_ values, we found an increased relative VO_2max_ in the VEG group, which was significantly different from that of the CON group (57.0 [53.7–60.3] mL/kg/min (VEG) vs. 51.6 [48.1–55.0] mL/kg/min (CON); *p* = 0.041, Cohen’s *d* = 1.675). Conclusions: These findings suggest that a short-term vegan diet does not adversely affect training-induced performance improvements and may be suitable for semi-professional soccer players.

## 1. Introduction

The relationship between diet and athletic performance has been a central focus in the field of sports science. It is a multifaceted association influenced by factors such as nutrient timing, energy balance, and the type and quality of the food consumed [1]. Nutrition is crucial for several functions, such as health, optimal athletic performance, and recovery, as it provides the body with the necessary building blocks to repair damaged tissues and support training adaptations [2,3]. Semi-professional athletes are defined as players competing at a regional level with regular training and match schedules, while receiving limited financial compensation. This distinguishes them from recreational athletes (non-competitive, leisure-based) and elite athletes (full-time professionals at the national or international level).

The research on sports nutrition specific to football has grown significantly [2]. Soccer is a physically demanding sport that requires high levels of strength, endurance, and agility with increasing demands, making it an interesting subject for researching the impact of diet on athletic performance [4]. Several studies have investigated the impact of different dietary interventions on soccer performance. However, the current research mainly investigates the influence of individual nutrients [5,6,7] and less specific diets, especially in recreational soccer, while data on elite soccer is rare [2].

In recent years, plant-based diets have gained popularity among athletes, as they have been shown to offer several health benefits, including improved cardiovascular function, reduced oxidative stress, and anti-inflammatory effects, which may enhance recovery and muscular adaptations [8,9,10,11]. Also, plant-based diets may positively influence mitochondrial function and the metabolic signaling pathways relevant to endurance and strength performance [11]. In addition to health reasons, sustainability aspects have also become increasingly important [10,12]. Also, the prevalence of vegetarians and vegans is growing in endurance sports [13], and vegan-friendly supplements are being increasingly explored as tools to optimize performance and support training needs [14]. However, the effects of a plant-based diet on athletic performance are not fully understood. Recent case reports have demonstrated that vegan athletes can still achieve top athletic performances [15,16,17]. Also, a few cross-sectional studies and one recent short-term intervention study on veganism in (strength) endurance sports showed no disadvantages in terms of quality of life, nutritional status, and exercise capacity [13,18,19,20,21,22,23,24]. While the results of these studies suggest that a vegan diet does not have any adverse effects on the performance of athletes, individual case reports and cross-sectional studies are not sufficient to definitively establish causal links between diet and performance. Moreover, the results of long-distance runners in recreational sports are hardly transferable to endurance athletes in elite sports, especially with the different nutrient requirements and utilization methods.

A further relevant issue concerning vegan diets that is often discussed is the risk of insufficient intake of certain macronutrients, such as protein and omega-3 fatty acids, as well as micronutrients, including vitamin B_12_, iron, zinc, calcium, and iodine [25]. These nutrients are especially critical for athletes due to their roles in oxygen transport, energy metabolism, immune function, and muscle repair.

Despite the growing interest in plant-based diets among athletes, there is limited research on the effects of a vegan diet on soccer performance. This unique pilot study aimed to examine the impact of an 8-week vegan diet on the nutritional status and exercise performance of male semi-professional soccer players. Based on the existing literature, the study was designed to test whether an 8-week vegan dietary intervention would have no adverse effects on nutritional status or physical performance in this athletic population.

## 2. Materials and Methods

### 2.1. Study Design and Participants

This intervention study was designed as a controlled, non-randomized, longitudinal pilot study. To investigate whether a vegan diet affects the nutritional status and exercise performance of semi-professional soccer players, one group of participants was switched to a vegan diet for 8 weeks (self-selected due to practical constraints during the competitive season and the voluntary nature of dietary changes). The study took place during the season, so the subjects went about their usual training.

The study was performed between June and September 2021. The investigation took place at the University Outpatient Clinic Potsdam. Ethical approval was provided by the Ethics Committee of the University Outpatient Clinic Potsdam (Potsdam, Germany). The study was conducted in accordance with the Declaration of Helsinki. In addition, the principles of “Good Clinical Practice” (GCP) were applied. All subjects gave written informed consent.

Subjects were recruited from the soccer department of the SV Babelsberg 03 sports club in Potsdam, Germany, which at that time played in the third German league. Due to the behavioral demands of a vegan diet and to ensure compliance and feasibility during the competitive season, the participants self-selected a dietary group. Randomization was therefore not considered feasible within the context of this pilot study.

The study subjects who performed one or more of their exercise tests on a bicycle ergometer instead of a treadmill—mainly due to training-related injuries (e.g., leg, foot, or knee injury)—were not excluded from the study but were omitted from the statistical analysis due to protocol deviation. In addition, subjects with incomplete performance data or statistical outliers (Q = 1%) were excluded based on a per-protocol analysis.

Ultimately, 16 study subjects completed the study. Performance data from 12 subjects and anthropometric data from 15 subjects were included in the statistical analysis. Subjects were selected based on the following inclusion criteria: male, age ≥ 18 years, no relevant primary disease, and active (semi-)professional player of the SV Babelsberg 03 (participating in regular football practice and games). The following criteria led to exclusion: vegan diet, vitamin B_12_ deficiency, any diagnosed food allergy, renal dysfunction, chronic metabolic disorder, and abnormal medical findings in the initial health inspection.

### 2.2. Study Procedure

The subjects in the VEG group followed a vegan diet for 8 weeks during ongoing training and matches, while the subjects of the CON group maintained their usual omnivorous diet. Prior to the dietary change, the participants in the VEG group and, if not primarily responsible for meal preparation, their household members, received a practical cooking session and written instructions to support adherence. Regular personal contact, including check-ins and follow-up calls, was maintained throughout the study to provide guidance, answer questions, and encourage compliance with the assigned diet. During the intervention period (t1 to t2), whole-food vegan and omnivore lunch meals were provided by a caterer to the vegan and control groups, respectively. The rest of the meals had to be self-prepared by the subjects. Dietary supplements were not provided.

**Figure 1:**
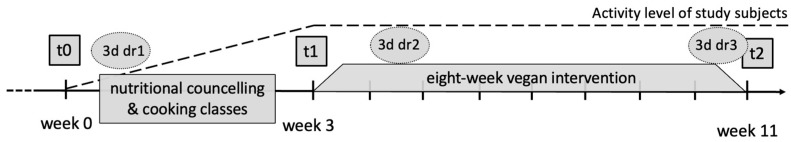
Timeline of the study procedure. t0: baseline; t1: start of intervention; t2: end of intervention; 3d dr 1–3: 3-day dietary record at t0–t2; dashed line: activity level of study subjects.

Examinations of nutritional status and exercise performance were conducted at three time points: during the inclusion examination (t0), after the start of the nutritional intervention (t1), and after the intervention period (t2) (Figure 1). On examination days, the participants were invited to the University Outpatient Clinic Potsdam (t0, t1, and t2).

#### 2.2.1. Assessment of Dietary Intake and Nutritional Biomarkers

To monitor dietary adherence, all participants completed 3-day food records at baseline (t0), mid-intervention (t1), and post-intervention (t2). At t0, both groups had their usual diet, since the intervention started afterward (Figure 1). The 3-day dietary records were checked for plausibility and completeness, and analyzed by trained nutritionists with the OptiDiet Basic Software (Version 6.0.0.001, GOE mbH, Linden, Germany). The foods were assigned to food groups, as described previously [26]. Ambiguities were clarified with the subjects if necessary. Food that was not available in the software was added.

Additionally, nutritional status was analyzed via venous blood samples that were taken after overnight fasting (12 h) at t0, t1, and t2. All micronutrient parameters described below were determined in an accredited and certified laboratory (Institut für medizinische Diagnostik Berlin-Potsdam GbR, IMD).

Biochemical analyses were performed on serum and EDTA blood samples. Lipid parameters, including total cholesterol, HDL, LDL, and triglyceride levels, were determined using photometric methods. Hemoglobin levels were measured using the SLS hemoglobin method, while ferritin and folic acid levels were analyzed via chemiluminescent microparticle immunoassays (CMIAs). Vitamin D levels were assessed using chemiluminescent immunoassays (CLIAs), whereas β-carotene, vitamin E, vitamin A, and vitamin B_2_ levels were quantified via high-performance liquid chromatography (HPLC). Magnesium levels were determined photometrically, while zinc concentrations were measured using inductively coupled plasma mass spectrometry (ICP-MS).

#### 2.2.2. Anthropometric Measurements

First, anthropometric measurements were carried out. Body weight (BW), fat mass (FM), fat-free mass (FFM), total body water (TBW), resting energy expenditure (REE), and total energy expenditure (TEE) were measured using a medical body composition analyzer (Seca^®^, Hamburg, Germany). Additionally, skinfold thickness (SKF) for the determination of FM percentage was measured using a Harpenden skinfold caliper (Harpenden, Baty, Burgess Hill, UK) according to the equations from Durnin and Womersley [27] and Parízková and Bůzková [28].

#### 2.2.3. Incremental Exercise Test

Second, an incremental exercise test was performed. To ensure that the participants could take part in the test, they consulted a physician before the test. All subjects performed a standardized incremental treadmill test (h/p/cosmos Pulsar 3p 4.0, Nussdorf-Traunstein, Germany) starting at 6 km/h, with an incremental increase of 2 km/h every 3 min until voluntary exhaustion. The subjects were asked to carry a turbine-type face mask (V2 Mask, Hans Rudolph Inc., Shawnee, KS, USA) that analyzed both tidal volume and ventilatory gases (VO_2_, volume of oxygen uptake). During the test, 15–20 μL of blood was drawn from the earlobe with a lancet and immediately transferred into a glucose/lactate hemolysis solution for the analysis of lactate concentrations (EKF BIOSEN S-line Lab, Barleben, Germany). Shortly before reaching exhaustion, all the subjects were verbally motivated to achieve maximum performance. The performance data was directly analyzed and the individual anaerobic threshold (IAT) was calculated for each subject (Ergonizer software, Version 5.10.4, Build 50, Freiburg i. Brsg., Germany). All measurements were carried out by a professional employee.

### 2.3. Data Analysis and Statistical Methods

The statistical analysis of the data was performed using GraphPad Prism 9.2.0 (GraphPad Software, San Diego, CA, USA) and SPSS Software (IBM SPSS Statistics 18.0; Chicago, IL, USA). As this was an exploratory pilot study and no prior data on VO_2max_ responses to a vegan diet assessed via a standardized incremental treadmill test were available, no formal sample size calculation was performed. The study was designed to estimate effect sizes (e.g., Cohen’s *d*) to support future adequately powered trials.

First, descriptive statistics (mean, SD, 95% confidence interval) were used to summarize the data from both study groups. Second, baseline subject characteristics were analyzed for significant differences between groups using two-way ANOVA and Pearson’s chi-squared test. Third, the data were analyzed for significant differences between the groups or time points using two-way ANOVA with repeated measures, with ‘dietary group’ (vegan vs. control) as the between-subject factor and time points (t0–t2) as the within-subject factor. Moreover, the effect sizes were quantified using Cohen’s *d*. Lastly, significant data were additionally corrected using a post hoc analysis, either using the Tukey method for within-subject comparisons or Šídák’s multiple comparisons correction for between-subject comparisons. Group differences in temporal delta were calculated using an unpaired *t*-test. Outliers were identified using the ROUT method with a false discovery rate (Q) of 1%. In cases with missing values, such as due to the elimination of outliers, a mixed-effects analysis (or ‘mixed model’) was conducted instead. The study participants with incomplete performance data or who deviated from the study protocol during one of their performance tests on the treadmill or cycle ergometer were excluded from the statistical analysis for that specific protocol. The results are shown as the means and 95% confidence intervals (CIs), and significance was assumed at *p* ≤ 0.05.

## 3. Results

The anthropometric and sociodemographic data at baseline are depicted in Table 1. The study groups were comparable in terms of age, body composition, education, and dietary supplement intake.

### 3.1. Dietary Intake and Nutritional Status

#### 3.1.1. Dietary Intake

The transition to a vegan diet was associated with a higher intake of legumes, nuts (*p* < 0.05), and plant-based milk and meat alternatives (VEG group; Table 2). Notably, the VEG group consistently exhibited higher mean consumption levels for vegetables (except t0), fruits (t1: *p* = 0.015), and grains across all three measurement time points. The consumption of convenience foods showed an increase in both groups at t2, with the VEG group surpassing the CON group in this regard (160 [32.6–287] g (VEG) vs. 89.8 [−52.4–232] g (CON)).

The variations in food consumption led to discernible distinctions in nutrient intake. Over the course of the study, energy and fat intake increased in both groups (Table 3), with slightly lower fat intake in the VEG group (32.2 to 36.6 En% vs. 35.2 to 40.1 En% in the CON group). Carbohydrate intake consistently registered higher levels in the VEG group, with a large effect size (t2: 46.2 [40.3–52.2] En% (VEG) vs. 37.6 [34.1–41.1] En% (CON); *p* = 0.036, Cohen’s *d* = 1.321). Conversely, protein intake was consistently lower in the VEG group at all time points.

In terms of micronutrients, we identified an overall augmented intake of vitamin A, vitamin E, vitamin K, folic acid, and vitamin B_12_ (attributable to supplementation, as detailed in Table S1), as well as magnesium and iron in the VEG group compared to the controls. Intriguingly, we observed an increased consumption of vitamin B_12_ supplements in the VEG group and vitamin C, calcium, and iodine supplements in the CON group (Table S1).

#### 3.1.2. Nutritional Status

Most of the biochemical parameters generally fell within the reference range for both groups across all the measurement time points (as depicted in Table 4 and Table 5). However, the TG levels in the CON group exceeded the reference value of 1.7 mmol/L at t2 (1.95 [−0.16–4.05] mmol/L). Further, the total cholesterol level decreased significantly in the VEG group during the intervention (−0.36 mmol/L, *p* = 0.012) (Table 4). There were no substantial changes in hematological parameters or vitamin and mineral blood concentrations in both groups during the course of the intervention (Table 5).

### 3.2. Anthropometrics and Exercise Performance

#### 3.2.1. Anthropometrics

Initially, both groups displayed comparable anthropometric data at baseline (Table 6). However, throughout the intervention, the VEG group exhibited a significant weight loss of −1.94 kg (*p* = 0.007). In contrast, the CON group showed a modest, albeit non-significant, decrease in body weight (−0.48 kg) (Table 6). Furthermore, the outcomes concerning body mass composition varied depending on the measurement methodology employed. The BIA data indicated no difference in mean fat mass (FM_b_) between the VEG (+0.40%) and CON (+0.68%) groups. In contrast, the results obtained from skinfold measurements (SKF) suggested a non-significant reduction in fat mass for both groups (VEG: −0.61%; CON: −0.27%). Moreover, it is noteworthy that the VEG group experienced a notable reduction in absolute fat-free mass (FFM_b_) by the conclusion of the intervention (−2.02 kg, *p* = 0.013), whereas the CON group did not exhibit a significant change in FFM_b_.

#### 3.2.2. Exercise Performance

**Figure 2:**
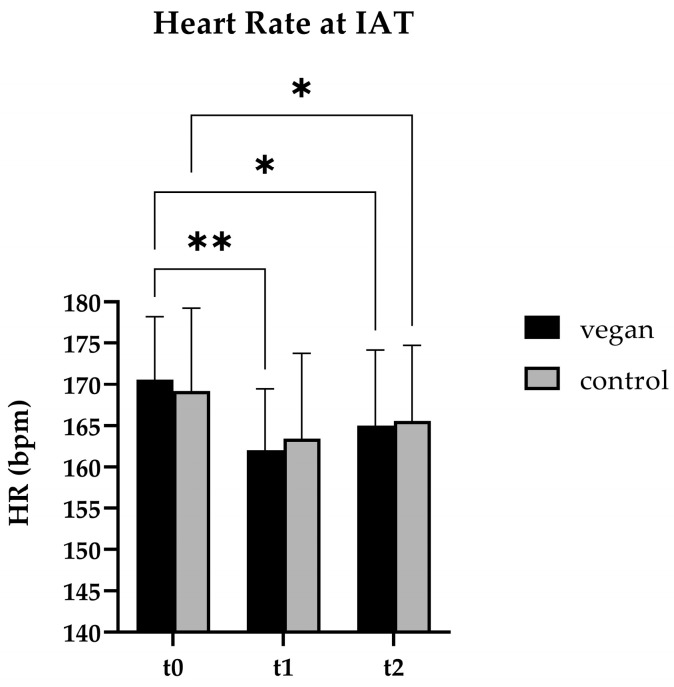
Heart rate (HR) at the individual anaerobic threshold (IAT) of the vegan (n = 7) and control (n = 5) subjects at baseline (t0), start of the intervention (t1), and end of the intervention (t2). The vegan (*p* = 0.034) and control groups (*p* = 0.038) significantly reduced their heart rate between t0 and t2. * *p* ≤ 0.05, ** *p* ≤ 0.01 (2-way ANOVA).

At baseline, the resting **heart rate** in the VEG group was higher than in the CON group, but it decreased significantly during the intervention (−15.4 bpm, *p* = 0.013). The IAT analysis at t2 demonstrated a significant decrease in heart rate (VEG: −5.57 bpm, *p* = 0.034; CON: −3.60 bpm, *p* = 0.038) for both groups (Figure 2), with no discernible differences in maximum heart rate.

Regarding **velocity**, both groups had significantly increased v_IAT_ values, but only the VEG group displayed a significant increase in v_max_ at t2 (+1.88 km/h, *p* = 0.013; Table 6).

**Figure 3:**
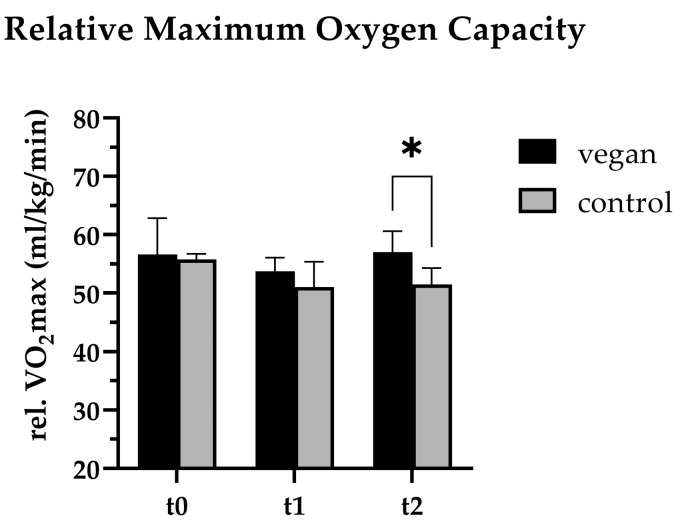
Relative maximum oxygen capacity (rel. VO_2max_) of the vegan (n = 7) and control (n = 5) study subjects at baseline (t0), the start of the intervention (t1), and the end of the intervention (t2). At t2, the VEG group had a significantly (*p* = 0.017) higher rel. VO_2max_ compared to the controls. * *p* ≤ 0.05 (2-way ANOVA).

While the absolute **VO_2max_** values remained relatively stable in the VEG group, the CON group experienced a notable reduction (−366 mL/min) during the intervention period, although this change did not reach statistical significance. Notably, the relative VO_2max_ in the VEG group was significantly higher than in the CON group at t2 (57.0 [53.7–60.3] ml/kg/min (VEG) vs. 51.6 [48.1–55.0] ml/kg/min (CON); *p* = 0.041, Cohen’s *d* = 1.675) (Table 6, Figure 3). Comparing the t0 and t2 VO_2max_ within the groups, there was no difference in the VEG group (+0.43 mL/kg/min) and only a slight reduction in the CON group (−4.24 mL/kg/min).

**Figure 4:**
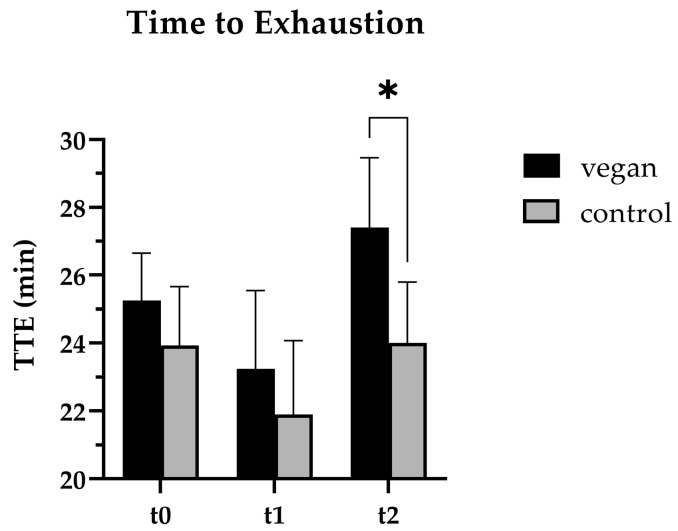
Time to exhaustion (TTE) of vegan (n = 7) and control (n = 5) subjects at baseline (t0), the start of the intervention (t1), and the end of the intervention (t2). At t2, the VEG group showed a significantly (*p* = 0.014, Cohen’s *d* = 1.740) longer TTE than the controls. * *p* ≤ 0.05 (2-way ANOVA).

The duration of treadmill running until exhaustion (time to exhaustion, **TTE**) was consistently longer in the VEG group at all time points, and was significant at t2 (27.4 [25.5–29.3] min (VEG) vs. 24.1 [21.8–26.2] min (CON); *p* = 0.039, Cohen’s *d* = 1.740) (Figure 4). Comparing the t0 and t2 TTE within the groups, there was a slight increase in the VEG group (+2.14 min) and no difference in the CON group (+0.08 min).

## 4. Discussion

This is the first study to examine the effect of a vegan diet on dietary intake, nutrient status, and exercise performance in semi-professional soccer players during the competitive season. Since there are no directly comparable intervention studies, our results were compared to data from investigations considering different types and intensities of sports (e.g., recreational athletes), cross-sectional studies, and omnivorous athletes.

### 4.1. Interpretation of Dietary Intake and Nutritional Outcomes

One of the aims of this study was to examine how a vegan dietary intervention affects food consumption, nutrient intake, and biochemical status of semi-professional soccer players. These findings could aid in formulating initial recommendations for this target group. Overall, our results suggest that a well-planned vegan diet in this context can ensure the supply of most nutrients.

While vegetarianism receives at least a modicum of attention in recommendations from professional societies such as the ACSM, IOC, and ISSN, veganism has received little to no attention [1,30]. Also, as these few existing recommendations for mainly high-performance athletes were only partially applicable to our study sample, the nutrient intake was compared with the intake recommendations of the German and Austrian Societies for Nutrition for the general population [29], even though they have not published any specific recommendations for vegan athletes. However, one recent work gave the first practicable recommendations for vegan athletes in the form of a VegPlate [31].

During the competition season, the participants completed almost daily training sessions of 60–90 min, and each player also had about 38–40 game days with higher physical exertion. Since this training volume is not classified as a high-performance sport but rather a higher activity level than recreational sports, a PAL index of 1.8 was chosen as the **energy** intake reference value. However, both groups had comparable energy intakes and neither group met the recommended energy intake of 3000–3100 kcal/day [29]. For team sports like football, the energy demands vary during the game and between the individual positions (striker, defender, goalkeeper, etc.), making it difficult to determine the energy expenditure. Therefore, it is recommended to have a higher energy intake to avoid performance deficits in football players and team sport athletes in general [32,33].

Although the VEG group participated in nutritional counseling and cooking classes before the intervention, our results suggest that some challenges, like meal planning and achieving the energy and nutrient needs, should be taught more effectively and athletes should be supervised more closely. However, this also applied to the CON group. Future studies should be standardized in terms of energy and maybe macronutrient intake for both groups.

The daily **carbohydrate** recommendation and intake for athletes depend on several factors such as the sport type, training intensity, gender, and external factors. The energy requirement for athletes is 55–60% or 3–8 g/kg BW carbohydrates for in-season training, so that athletes can benefit from filled glycogen stores [1,2,34]. Although the VEG group showed a higher carbohydrate intake compared to the CON group (t2: 46.2 [40.3–52.2] En% (VEG) vs. 37.6 [34.1–41.1] En% (CON); *p* = 0.036, Cohen’s *d* = 1.321), both groups did not meet the recommendations. Our results are partially consistent with previous research since numerous studies have shown that high amounts of carbohydrates can be achieved with a plant-based diet, which were also significantly higher and within the recommendations in vegan athletes compared to omnivores [19,35,36].

**Fat** is an important source of energy and is also crucial for various metabolic processes, especially in the form of essential fatty acids. In our study, the fat intake exceeded the recommended amount of 30% of the energy intake [30] in both groups. Ketogenic low-carbohydrate, high-fat diets are not recommended for soccer players due to insufficient data, but amounts of up to 42 En% fat are still considered safe [2,37]. In accordance with existing literature, the VEG group showed a more favorable blood fatty acid profile compared to the CON group [38].

The **protein** recommendations for athletes are still being discussed [10,39,40] and range between 1.2 and 2.0 g/kg BW [1,41,42]. As expected, the VEG group consumed less protein compared to the controls. While the VEG group reached the protein intake recommendation for healthy men [29], they did not reach the levels recommended for athletes. A previous cross-sectional study found that omnivores, vegetarians, and vegans could all reach the protein intake reference values for recreational athletes [19]. Recent studies showed that professional soccer players can even exceed protein requirements [43]. For vegans, it is essential to incorporate a variety of plant-based protein sources such as legumes, nuts, and whole grains to enhance protein quality [42]. In the short term, closer monitoring by nutrition experts may be beneficial.

**Micronutrient** intake was generally sufficient for most nutrients, except for iodine in both groups, calcium and zinc in the VEG group, and potassium and folate in the CON group. Overall, the micronutrient status was adequate in both groups. Specifically, the 25(OH)D concentrations were within the normal range, though in the lower part of the normal range (110 [84.1–136] nmol/L (VEG) vs. 92.4 [67.0–118] nmol/L (CON); *p* = 0.599, Cohen’s *d* = 0.577). As the vitamin D intake was similarly insufficient in both groups, the vitamin D supply seemed to have been covered by endogenous synthesis (since the study occurred in the summer) and was less dependent on dietary sources, as was observed previously in outdoor endurance athletes [20].

Iron is a potentially critical nutrient in plant-based diets and iron deficiency can negatively affect athletic performance [44]. In our study, dietary iron intake, as well as hemoglobin and ferritin concentrations, remained within the reference ranges in both groups at the end of the intervention (ferritin levels at t2: 133 [92.5–189] pmol/L (VEG) vs. 177 [124–230] pmol/L (CON); *p* = 0.350, Cohen’s *d* = 0.842). The ferritin levels tended to decrease from t0 to t2, possibly due to the increased physical demands following the summer break before t0. These findings are in line with previous studies comparing ferritin levels in vegan recreational athletes to those of lacto-ovo-vegetarians and omnivores [20]. However, it is important to highlight that female athletes following a vegan diet may face a higher risk of having an inadequate iron supply [45,46,47].

Contrary to our expectations, folate and vitamin C intake did not increase, while zinc and calcium intake remained relatively stable instead of decreasing as anticipated. This can likely be attributed to slightly lower fruit and vegetable consumption during the intervention and high baseline intake at the study’s start. Accordingly, there were also only minimal changes in blood levels.

Supplement intake (both individual and multivitamin/mineral complexes) was relatively low in both groups. Although the use of supplements and sports nutrition seems to be widespread in soccer [48], supplementation should always be individualized, with guidance from nutrition experts. The Union of European Football Associations (UEFA) expert group strongly recommends the “food first principle” as the foundation of dietary strategies [2].

### 4.2. Interpretation of Anthropometrics and Exercise Performance

In this pilot study, we observed a significant reduction in **body weight** among the participants following a vegan diet after 8 weeks (−1.94 kg, *p* = 0.007), whereas no significant reduction was found in the control group (−0.37 kg). Although both groups participated in the same training schedule and match routines, only the vegan group showed a statistically significant reduction in body weight. Therefore, dietary differences are likely to have contributed to this observation.

The weight loss may also be partly attributed to the increased physical workload during the 8-week training and competition phase, as the participants returned from a three- to four-week summer break without structured football training. Interestingly, the energy intake in both groups remained below the calculated total energy expenditure and even decreased in the control group. Energy intake was calculated based on self-reported food intake and therefore could have been underestimated. Nevertheless, it is well documented that vegans generally show a lower body weight compared to omnivores due to the lower energy density of a vegan diet [49,50,51], while data on vegan athletes have shown mixed results [18,23].

**Body composition** changes in the VEG and CON groups were assessed using bioelectrical impedance analysis (BIA) and skinfold thickness (SKF) measurements. The fat mass values in both groups were within the typical range for soccer players (8–13%), as previously reported using DXA, which varies depending on season and playing position [52,53]. The VEG group showed a significant reduction in FFM_b_ (−2.02 kg, *p* = 0.013) compared to the smaller, non-significant reduction in the controls (−0.89 kg). This finding contrasts with earlier studies reporting increases in fat-free mass up to mid-season [54,55]. The validity of the BIA data has been discussed and was found to be imprecise in very lean or obese individuals [56,57]. Nevertheless, the significant weight loss observed in the VEG group may indicate a reduction in fat-free mass, which contrasts with the findings from previous studies [18,58]. One possible explanation could be the lower mean protein intake in the VEG group (1.13 [0.81–1.44] g/kg BW (VEG) vs. 1.68 [0.81–2.55] g/kg BW (CON)) which—although exceeding the general recommendation (0.8 g/kg BW)—was below the recommended range for athletes (1.2–2.0 g/kg BW). Recent evidence suggests that well-planned plant-based diets can support muscle maintenance and training adaptations as well as omnivorous diets, provided that the protein intake is sufficient (~1.6 g/kg BW) [59,60]. However, in this study, the observed intake may have been insufficient to meet the increased demands of the competitive training phase. In addition to quantity, the protein quality, amino acid composition, and distribution across meals are key factors influencing muscle protein synthesis in vegan athletes [61,62]. These findings underline the importance of individualized nutritional counseling and careful dietary planning when implementing vegan diets in athletic populations.

During the intervention, the **heart rate** at IAT in both groups decreased significantly. The results showed a positive adaptation of the heart to increased exercise frequency and intensity [63]. The short-term vegan diet did not negatively affect or interfere with the positive training adaptations of the increased workload experienced by the soccer team throughout the season.

Measuring **lactate** concentrations in the blood during an incremental exercise test can provide valuable information about energy metabolism. As physical activity intensity increases, the body relies more on anaerobic energy production, leading to higher lactate synthesis. Lactate accumulation is associated with muscular fatigue. The point at which it accumulates rapidly has been defined as the individual anaerobic threshold (IAT), which occurs at higher percentages of the maximal workload in trained individuals [64,65]. In our study, both the VEG and CON groups exhibited a slight reduction in blood lactate levels at the IAT (VEG: −0.63 mmol/L; CON: −0.08 mmol/L), without significant differences between the groups, suggesting no relevant changes due to the different diets.

Notably, the vegan group consistently showed significantly higher maximum lactate values compared to the control group, which complicates the interpretation of the results. However, these higher maximum lactate values were likely attributable to the VEG group’s increased maximal running speed and time to exhaustion at t0, t1, and t2. A reduction in carbohydrate intake during the intervention could also influence lactate metabolism, given its reliance on glycogen stores. Nonetheless, previous cross-sectional data indicate that lactate concentrations in vegan recreational athletes, despite their higher carbohydrate intake, were comparable to those in lacto-ovo-vegetarians and omnivores [18]. Various factors, including prior physical activity, hydration levels, and caffeine intake, can impact lactate kinetics during incremental exercise, and individuals’ sex and genetic background seem to be of greater relevance than diet alone [66,67].

Regarding **running velocity**, both groups similarly improved their values at the intensity associated with the individual anaerobic threshold (v_IAT_) throughout the study (VEG: +1.21 km/h, *p* = 0.002; CON: +1.42 km/h, *p* = 0.028). Additionally, both groups increased their maximum running velocity (v_max_) over time, with only the VEG group showing a significant improvement during the intervention period (+1.88 km/h, *p* = 0.013). Previous studies did not observe any differences in peak power between vegan and omnivorous athletes [18,68]. Neither group reached the recommended daily carbohydrate intake of ≥ 55% of daily energy to adequately replenish glycogen stores [69]. However, the VEG group had a higher carbohydrate intake. It can be assumed that the VEG group possessed higher glycogen levels due to their higher carbohydrate consumption, which might explain their higher lactate concentrations and greater v_max_ compared to the CON group.

Another common parameter used to assess athletic performance is **VO_2max_**. In the VegInSoc study, the relative VO_2max_ was significantly higher in the VEG group at t2 (57.0 [53.7–60.3] mL/kg/min (VEG) vs. 51.6 [48.1–55.0] mL/kg/min (CON); *p* = 0.041, Cohen’s *d* = 1.675). These results could be explained by the decreased body weight in the VEG group, as similar results were observed in other studies, including differences in relative, but not absolute, VO_2max_ between vegan/vegetarian and omnivorous athletes [68,70].

To evaluate endurance capacity, we also assessed the **TTE** and found that the VEG group improved their TTE by 2.15 min between baseline and t2, whereas almost no change was observed in the CON group. The TTE was longer in the VEG group throughout the study, but the difference only became statistically significant at t2 (27.4 [25.5–29.3] min (VEG) vs. 24.1 [21.8–26.2] min (CON); *p* = 0.039, Cohen’s *d* = 1.740). Only a few studies in the literature comparing the performance capacity of vegans and omnivores have provided information about TTE, with contradicting results [58,71]. The higher carbohydrate content in a vegan diet compared to an omnivorous diet may have facilitated a longer maximum working capacity. However, the carbohydrate consumption during the study cannot explain the improvements in TTE in the groups and could be exercise-induced as a result of the increased training and competition.

To our knowledge, the VegInSoc study is the first study to evaluate the effect of a vegan diet intervention on the food intake, nutrient status, and performance of semi-professional soccer players. The key strengths of the study include the practical design under real-world training conditions and the comprehensive analysis of dietary, performance, and health-related parameters. However, the limitations include the small sample size, non-randomized group allocation, and the short intervention period of 8 weeks, which limits the generalizability and long-term applicability of the findings. The occasional negative values for supplement-based nutrient intake (Table S1) likely resulted from incomplete product information or software limitations and are considered artefacts. Furthermore, it would be interesting to investigate 24 h dietary recalls before the performance tests. In addition, nutrition should be considered separately on match day, as nutrient timing plays a crucial role at professional levels. Also, investigations with different stages of age and performance levels would be of great interest.

## 5. Conclusions

In summary, this pilot study suggests that a well-planned, short-term vegan diet does not impair exercise performance or nutritional status in semi-professional soccer players. These findings indicate that a plant-based diet may be a feasible option for this group of athletes. However, due to the exploratory nature of the study and its limitations—including the small sample size and non-randomized design—further long-term intervention studies with larger, randomized cohorts are needed to validate these preliminary results and better control for potential confounding factors.

## Figures and Tables

**Table 1:** Characteristics of the study population at baseline (t0).

Measure	VEG (n = 9)	CON (n = 6)	*p*-Value
Age (y)	23.4 [19.9–27.9]	24.0 [19.4–28.6]	0.819
Anthropometric data			
BMI (kg/m^2^)	24.0 [22.6–25.4]	23.7 [23.0–24.4]	0.699
REE (kcal/d)	1787 [1710–1864]	1793 [1697–1889]	0.916
TEE (kcal/d)	3574 [3420–3729]	3586 [3393–3778]	0.916
FFM_b_ (kg)	69.1 [65.0–73.3]	68.9 [61.3–76.5]	0.941
FM_b_ (%)	10.5 [5.87–15.2]	14.0 [10.8–17.3]	0.271
FM_c_ (%)	11.4 [10.0–12.8]	13.0 [11.4–14.6]	0.095
TBW (%)	63.0 [57.8–68.3]	62.3 [60.0–64.5]	0.795
Sociodemographic data (n [%])	VEG (n = 8)	CON (n = 3)	
Education			0.245
High school diploma	6 [75]	1 [33]	
Technical college	1 [12.5]	0 [0]	
Secondary school	1 [12.5]	1 [33]	
Main school	0 [0]	1 [33]	
Occupation			0.458
Full time	2 [25]	2 [33]	
Part time	2 [25]	1 [67]	
In training	1 [12.5]	0 [0]	
Not working	3 [37.5]	0 [0]	
Tobacco Smoking			0.521
Never	7 [87.5]	0 [0]	
Occasionally	1 [12.5]	3 [100]	
Daily	0 [0]	0 [0]	
Alcohol Consumption			0.782
Never	2 [25]	1 [33]	
Occasionally	6 [75]	2 [67]	
Daily	0 [0]	0 [0]	

VEG: vegan group; CON: control group; BMI: body mass index; REE: resting energy expenditure; TEE: total energy expenditure; FFM_b_/FM_b_: fat free mass/fat mass measured with BIA; FM_c_: fat mass measured with calipers; TBW: total body water. Group differences were assessed using independent samples *t*-test for metric data and Pearson’s chi-squared test for ordinal data.

**Table 2:** Intake of food groups during the intervention.

Food Group (g)	Group	t0 Mean [95% CI]	t1 Mean [95% CI]	t2 Mean [95% CI]	Δ(t2 − t1)	Δ(t2 − t0)
Vegetables	VEG	248 [109–386]	424 [185–663]	216 [97.9–334]	−208	−31.7
	CON	275 [38.7–511]	143 [3.15–283]	118 [6.86–229]	−24.8	−157
	*p*-value	0.992	0.095	0.404	0.140	0.176
	Cohen’s *d*	0.150	1.074	0.722		
Fruit	VEG	363 [195–531]	369 [197–541]	267 [78.8–455]	−102	−96.5
	CON	92.2 [−34.3–219]	69.6 [−51.8–191]	51.9 [−34.6–138]	−17.7	−40.3
	*p*-value	**0.025**	**0.015**	0.097	0.341	0.521
	Cohen’s *d*	1.444	1.565	1.055		
Grains	VEG	414 [273–555]	545 [348–743]	441 [310–572]	−105	+27.1
	CON	349 [113–584]	417 [212–622]	396 [207–584]	−21.3	+47.4
	*p*-value	0.908	0.626	0.946	0.542	0.868
	Cohen’s *d*	0.353	0.557	0.275		
Potatoes	VEG	105 [37.9–173]	60.2 [6.84–114]	76.9 [28.1–126]	+16.7	−28.5
	CON	114 [40.4–187]	133 [–33.8–300]	92.3 [−71.8–256]	−40.6	−21.3
	*p*-value	0.996	0.669	0.994	0.278	0.918
	Cohen’s *d*	0.104	0.756	0.166		
Legumes	VEG	29.4 [−16.2–74.9]	40.4 [2.66–78.2]	45.8 [−3.96–95.6]	+5.37 *	+16.5
	CON	0 [0–0]	4.54 [−8.07–17.2]	0 [0–0]	−4.54	0
	*p*-value	0.439	0.178	0.187	0.796	0.713
	Cohen’s *d*	0.607	0.885	0.866		
Nuts	VEG	41.2 [−25.0–107]	26.2 [8.44–44.0]	43.1 [18.0–68.2]	+16.9 *	+1.91
	CON	4.32 [−3.87–12.5]	0 [0–0]	2.73 [−4.84–10.3]	+2.73	−1.59
	*p*-value	0.555	**0.028**	**0.017**	0.058	0.929
	Cohen’s *d*	0.523	1.388	1.503		
Plant-based spreads	VEG	6.37 [−1.68–14.4]	4.00 [−0.95–8.95]	11.4 [−0.52–23.4]	+5.07	+7.44
	CON	0 [0–0]	0 [0–0]	0 [0–0]	0	0
	*p*-value	0.284	0.269	0.166	0.409	0.544
	Cohen’s *d*	0.746	0.761	0.901		
Oils and fats	VEG	19.3 [1.23–37.4]	23.8 [4.65–42.9]	29.9 [16.7–43.1]	+6.09	+21.0
	CON	19.2 [−3.27–41.8]	14.7 [−4.08–33.4]	23.4 [5.23–41.6]	+8.74	+4.15
	*p*-value	>0.999	0.795	0.854	0.819	0.528
	Cohen’s *d*	0.003	0.413	0.396		
Beverages	VEG	3260 [2217–4304]	2691 [1904–3479]	3113 [2580–3646]	+422	−147
	CON	2098 [403–3793]	2504 [919–4088]	2235 [137–4333]	−267	+137
	*p*-value	0.414	0.990	0.685	0.159	0.622
	Cohen’s *d*	0.855	0.168	0.779		
Milk and milk products	VEG	81.4 [28.2–135]	0 [0–0]	0 [0–0]	0	**−** 81.4 *
	CON	290 [237–343]	221 [31.7–410]	181 [4.72–357]	−40.0	−109
	*p*-value	**<0.001**	**0.092**	0.133	0.367	0.573
	Cohen’s *d*	3.379	2.511	2.208		
Milk alternatives	VEG	128 [16.8–239]	255 [51.4–459]	229 [25.3–433]	−25.6	+102
	CON	62.1 [−110–234]	80.9 [−93.5–255]	31.1 [−55.2–117]	−49.8	−31.0
	*p*-value	0.811	0.351	0.173	0.890	0.100
	Cohen’s *d*	0.461	0.754	0.900		
Meat	VEG	119 [28.9–209]	0 [0–0]	0 [0–0]	0	**−** 119 *
	CON	110 [24.4–196]	268 [48.9–487]	263 [134–391]	−5.61	+152
	*p*-value	0.998	0.080	**0.014**	0.857	**0.002**
	Cohen’s *d*	0.084	2.631	4.378		
Fish	VEG	31.4 [−8.84–71.7]	0 [0–0]	0 [0–0]	0	−31.4
	CON	37.3 [−30.0–105]	33.5 [−23.4–90.3]	0 [0–0]	−33.4	−37.3
	*p*-value	0.997	0.444	–	**0.043**	0.846
	Cohen’s *d*	0.111	1.265	n.c. ^1^		
Eggs	VEG	28.6 [4.43–52.7]	0 [0–0]	0 [0–0]	0	−28.6
	CON	36.9 [−15.6–89.5]	21.7 [−35.4–78.9]	26.7 [−29.7–83.1]	+4.93	−10.3
	*p*-value	0.976	0.726	0.594	0.820	0.486
	Cohen’s *d*	0.237	0.818	1.017		
Meat alternatives	VEG	13.3 [−13.3–39.9]	42.0 [5.20–78.8]	83.4 [16.3–151]	+41.4	+70.1
	CON	2.12 [−3.77–8.02]	5.72 [−4.01–15.5]	7.55 [−5.62–20.7]	+1.83	+5.43
	*p*-value	0.003	0.006	0.004	0.259	0.126
	Cohen’s *d*	0.393	0.922	1.059		
Convenience meals	VEG	63.0 [0.566–126]	53.0 [3.86–102]	160 [32.6–287]	+107	+96.8
	CON	50.8 [−14.0–116]	50.6 [−37.8–139]	89.8 [−52.4–232]	+39.1	+39.0
	*p*-value	0.982	>0.999	0.752	0.413	0.512
	Cohen’s *d*	0.168	0.036	0.466		
Sweets	VEG	30.1 [3.24–56.9]	52.0 [9.49–94.5]	29.5 [6.50–52.5]	−22.5	−0.57
	CON	95.8 [4.40–187]	86.1 [−16.2–188]	42.5 [11.0–74.0]	−43.6	−53.3
	*p*-value	0.315	0.824	0.795	0.553	0.119
	Cohen’s *d*	1.284	0.520	0.456		
Other	VEG	19.8 [−8.07–47.6]	26.8 [−1.84–55.4]	22.7 [−5.22–50.5]	−4.12	+2.89
	CON	84.5 [−23.3–192]	48.7 [−46.7–144]	51.9 [−41.6–145]	+3.24	−32.6
	*p*-value	0.438	0.923	0.834	0.364	0.176
	Cohen’s *d*	1.112	0.407	0.556		

VEG: vegan group (n = 9); CON: control group (n = 5); n.c.: not calculable. Group differences at the same time point were assessed using 2-way ANOVA and a connected post hoc analysis. Group differences in temporal delta were calculated using an unpaired *t*-test. Differences between time points within the VEG and CON groups were calculated separately (2-way ANOVA) and are reported as *p*-values in each case (* *p* < 0.05). Effect sizes are reported as Cohen’s *d* for between-group comparisons. ^1^ Cohen’s *d* could not be calculated due to the zero pooled standard deviation in both groups. Data shown in bold indicate significant values.

**Table 3:** Intake of energy, and macro- and micronutrients during the intervention (including supplements).

Measure and Reference Value	Group	t0 Mean [95% CI]	t1 Mean [95% CI]	t2 Mean [95% CI]	Δ(t2 − t1)	Δ(t2 − t0)
Energy (kcal)	VEG	2488 [1879–3096]	2507 [1846–3167]	2684 [2022–3346]	+177	+196
3000–3100 ^a^	CON	2583 [2007–3160]	2842 [1591–4092]	2606 [1330–3882]	−236	+22.8
	*p*-value	0.990	0.908	0.999	0.094	0.614
	Cohen’s *d*	0.137	0.368	0.085		
Macronutrients						
Carbohydrates (En%)	VEG	48.0 [44.2–51.8]	49.2 [44.9–53.5]	46.2 [40.3–52.2]	−2.98	−1.74
>50	CON	43.4 [35.1–51.7]	39.5 [32.1–41.1]	37.6 [34.1–41.1]	−1.92	−5.78
	*p*-value	0.531	0.054	**0.036**	0.756	0.320
	Cohen’s *d*	0.822	1.682	1.321		
Fat (En%)	VEG	32.2 [28.1–36.3]	33.7 [28.5–38.9]	36.6 [30.2–42.9]	+2.85	+4.36
30	CON	35.2 [27.8–42.6]	37.0 [28.1–45.9]	40.1 [35.8–44.3]	+3.04	+4.87
	*p*-value	0.763	0.811	0.644	0.965	0.890
	Cohen’s *d*	0.535	0.476	0.496		
EPA (mg)	VEG	72.1 [12.1–132]	30.1 [−33.7–93.8]	1.79 [0.29–3.30]	−28.3	−70.3
	CON	438 [−316–1192]	185 [14.4–355]	34.0 [13.7–54.3]	−151	−404
	*p*-value	0.578	0.179	**0.034**	0.067	0.124
	Cohen’s *d*	1.027	1.485	3.371		
DHA (mg)	VEG	170 [29.2–311]	42.6 [−25.6–111]	9.19 [−6.28–24.7]	−33.4	−161
	CON	671 [−288–1632]	235 [−62.5–533]	40.8 [−1.79–83.4]	−194	−630
	*p*-value	0.529	0.383	0.299	0.097	0.089
	Cohen’s *d*	1.066	1.232	1.228		
Protein (g/kg BW)	VEG	1.26 [0.99–1.53]	1.00 [0.71–1.29]	1.13 [0.81–1.44]	+0.13	−0.14
0.8	CON	1.48 [1.19–1.78]	1.85 [0.93–2.77]	1.68 [0.81–2.55]	−0.18	+0.20
	*p*-value	0.791	**0.007**	0.119	**0.038**	0.117
	Cohen’s *d*	0.690	1.614	1.052		
Fiber (g)	VEG	36.7 [21.8–51.5]	51.1 [36.2–65.9]	53.3 [38.6–68.0]	+2.27	+16.7 **
≥30	CON	28.0 [17.9–38.9]	32.6 [17.1–48.3]	25.4 [4.53–46.4]	−7.18	−2.51
	*p*-value	0.602	0.151	0.057	0.154	**0.003**
	Cohen’s *d*	0.526	1.060	1.513		
Vitamins						
A (RE, μg)	VEG	1139 [461–1817]	1167 [415–1919]	1480 [748–2212]	+313	+341
850	CON	1245 [643–1846]	962 [412–1512]	1188 [860–1517]	+226	−56.3
	*p*-value	0.989	0.937	0.794	0.882	0.423
	Cohen’s *d*	0.137	0.244	0.368		
D (μg)	VEG	12.3 [−9.11–33.8]	6.23 [−1.30–13.8]	13.6 [−7.64–13.8]	+7.41	+1.31
20	CON	5.15 [−0.54–10.8]	16.4 [−10.4–43.2]	18.1 [−14.0–50.1]	+1.66	+12.9
	*p*-value	0.852	0.744	0.988	0.748	0.360
	Cohen’s *d*	0.313	0.686	0.163		
E (mg)	VEG	20.0 [9.92–30.2]	29.9 [17.3–45.5]	32.5 [18.1–46.8]	+2.55	+12.4
15	CON	17.7 [9.46–26.0]	24.7 [11.0–38.5]	20.6 [0.72–40.4]	−4.16	+2.82
	*p*-value	0.965	0.873	0.560	0.142	0.211
	Cohen’s *d*	0.201	0.349	0.668		
K (μg)	VEG	157 [95.1–219]	172 [100–244]	218 [148–288]	+45.6	+60.5
70	CON	178 [−22.1–377]	189 [29.5–348]	116 [4.28–227]	−73.2	−62.0
	*p*-value	0.992	0.993	0.207	0.081	0.075
	Cohen’s *d*	0.178	0.156	1.130		
B_1_ (mg)	VEG	2.20 [1.13–3.27]	2.13 [0.97–3.28]	2.45 [1.40–3.50]	+0.32	+0.25
1.2–1.3	CON	1.76 [0.78–2.73]	3.15 [0.35–5.95]	2.58 [−0.13–5.30]	−0.57	+0.83
	*p*-value	0.842	0.781	0.999	0.174	0.335
	Cohen’s *d*	0.364	0.573	0.080		
B_2_ (mg)	VEG	1.83 [1.00–2.67]	1.78 [0.77–2.79]	1.56 [0.66–2.45]	−0.23	−0.28
1.4	CON	2.42 [1.34–3.50]	3.38 [0.32–6.43]	2.73 [−0.37–5.83]	−0.65	+0.31
	*p*-value	0.650	0.547	0.745	0.404	0.493
	Cohen’s *d*	0.577	0.896	0.682		
Niacin (mg)	VEG	35.2 [30.3–40.1]	30.2 [20.3–40.1]	25.0 [17.8–32.1]	−5.26	−10.3 *
15–16	CON	42.9 [31.8–54.1]	55.0 [23.5–86.5]	50.5 [21.2–79.9]	−4.50	+7.56
	*p*-value	0.361	0.258	0.199	0.896	0.075
	Cohen’s *d*	1.050	1.376	1.637		
Pantothenic acid (mg)	VEG	7.19 [4.00–10.4]	7.09 [2.96–11.2]	6.29 [3.07–9.51]	−0.80	−0.90
5	CON	8.93 [3.73–14.1]	12.0 [0.41–24.4]	10.2 [−2.51–22.9]	−1.82	+1.24
	*p*-value	0.858	0.728	0.839	0.600	0.414
	Cohen’s *d*	0.415	0.677	0.569		
B_6_ (mg)	VEG	2.53 [158–3.47]	1.98 [1.27–2.69]	2.25 [1.72–2.79]	+0.27	−0.28
1.6	CON	2.39 [1.22–3.56]	3.66 [0.56–6.76]	3.29 [−0.09–6.66]	−0.38	+0.90
	*p*-value	0.994	0.508	0.831	0.325	0.180
	Cohen’s *d*	0.120	1.033	0.621		
Biotin (μg)	VEG	66.4 [46.1–86.7]	70.0 [44.1–95.9]	70.7 [50.7–90.8]	+0.74	+4.36
40	CON	82.3 [45.3–119]	72.3 [−3.48–148]	68.5 [−15.0–152]	−3.76	−13.7
	*p*-value	0.727	>0.999	>0.999	0.684	0.448
	Cohen’s *d*	0.575	0.051	0.050		
Folic acid (μg)	VEG	415 [258–572]	401 [282–521]	363 [221–505]	−38.1	−51.5
300	CON	413 [178–648]	378 [139–617]	276 [−6.51–559]	−102	−137
	*p*-value	>0.999	0.994	0.866	0.408	0.303
	Cohen’s *d*	0.007	0.139	0.436		
B_12_ (μg)	VEG	23.6 [−19.2–66.3]	83.9 [−52.3–220]	84.0 [−51.9–220]	+0.21	+60.4
4.0	CON	6.80 [2.93–10.7]	75.9 [−119–271]	8.81 [0.25–17.4]	−67.1	+2.01
	*p*-value	0.776	>0.999	0.558	0.377	0.220
	Cohen’s *d*	0.369	0.046	0.521		
C (mg)	VEG	213 [85.2–341]	278 [123–433]	199 [90.5–307]	−78.9	−14.4
110	CON	362 [−246–970]	290 [−255–834]	327 [−339–993]	+37.2	−35.2
	*p*-value	0.904	>0.999	0.948	0.054	0.680
	Cohen’s *d*	0.474	0.039	0.387		
Minerals						
K (g)	VEG	3.63 [2.60–4.65]	4.02 [2.65–5.38]	4.05 [2.84–5.26]	+0.04	+0.42
4.0	CON	3.65 [2.96–4.34]	3.68 [1.77–5.59]	3.21 [1.30–5.11]	−0.48	−0.44
	*p*-value	>0.999	0.978	0.730	0.320	0.068
	Cohen’s *d*	0.019	0.196	0.543		
Ca (mg)	VEG	1009 [715–1303]	964 [686–1242]	907 [602–1212]	−56.8	−102
1000–1200	CON	1294 [866–1722]	1385 [557–2213]	1281 [427–2134]	−104	−13.2
	*p*-value	0.463	0.568	0.672	0.885	0.639
	Cohen’s *d*	0.770	0.867	0.729		
Mg (mg)	VEG	544 [355–732]	745 [472–1019]	741 [492–990]	−4.12	+198
350	CON	545 [321–770]	581 [248–914]	486 [103–868]	−95.6	−59.7
	*p*-value	>0.999	0.728	0.447	0.450	0.055
	Cohen’s *d*	0.007	0.499	0.802		
Fe (mg)	VEG	18.4 [10.7–26.2]	28.7 [14.5–42.8]	27.5 [15.9–30.1]	−1.18	+9.07
11	CON	15.9 [11.1–20.7]	21.0 [11.4–30.7]	16.8 [5.15–28.5]	−4.21	+0.94
	*p*-value	0.885	0.658	0.340	0.369	0.248
	Cohen’s *d*	0.299	0.487	0.795		
Zn (mg)	VEG	14.3 [9.31–19.2]	13.1 [7.13–19.0]	13.8 [8.54–19.0]	+0.72	−0.48
14 ^b^	CON	14.2 [9.46–19.0]	21.1 [6.02–36.2]	19.2 [5.81–32.7]	−1.88	+5.00
	*p*-value	>0.999	0.544	0.722	0.205	0.181
	Cohen’s *d*	0.004	0.855	0.653		
I (μg)	VEG	104 [60.3–147]	86.5 [61.6–111]	85.4 [47.8–123]	−1.01	−18.1
200	CON	154 [53.4–254]	174 [62.3–287]	126 [−3.63–255]	−48.9	−28.2
	*p*-value	0.599	0.257	0.837	0.179	0.785
	Cohen’s *d*	0.765	1.505	0.555		

VEG: vegan group (n = 9); CON: control group (n = 5); RE: retinol equivalents; dietary intake included supplement intake. Reference values of the German and Austrian Nutrition Societies (Deutsche und Österreichische Gesellschaften für Ernährung) [29]. ^a^ Physical activity level was estimated to be 1.8; ^b^ with medium level of phytate intake. Group differences at each time point were assessed using 2-way ANOVA and a connected post hoc analysis. Group differences in temporal delta were calculated using an unpaired *t*-test. Differences between time points within the VEG and CON groups were calculated separately (2-way ANOVA) and are reported as *p*-values in each case (* *p* < 0.05; ** *p* < 0.01). Effect sizes are reported as Cohen’s *d* for between-group comparisons. Data shown in bold indicate significant values.

**Table 4:** Blood lipid level of the study groups at all measurement time points.

Parameter and Upper/Lower Reference Value	Group	t0 Mean [95% CI]	t1 Mean [95% CI]	t2 Mean [95% CI]	Δ(t2 − t1)	Δ(t2 − t0)
Total cholesterol (mmol/L) <5.17 mmol/L	VEG	3.77 [3.30–4.24]	3.83 [3.40–4.26]	3.41 [2.95–3.87]	−0.42	−0.36 *
	CON	4.33 [3.26–5.39]	4.58 [3.29–5.88]	4.46 [3.57–5.35]	−0.13	+0.13
	*p*-value	0.605	0.500	0.087	0.370	**0.021**
	Cohen’s *d*	0.702	0.858	1.485		
HDL cholesterol (mmol/L) >1.55 mmol/L	VEG	1.49 [1.33–1.65]	1.44 [1.30–1.58]	1.42 [1.22–1.62]	−0.02	−0.07
	CON	1.48 [1.15–1.81]	1.55 [1.15–1.96]	1.54 [1.07–2.01]	−0.02	+0.06
	*p*-value	0.999	0.889	0.930	0.500	0.092
	Cohen’s *d*	0.068	0.412	0.333		
LDL cholesterol (mmol/L) <2.97 mmol/L	VEG	2.16 [1.63–2.68]	2.22 [1.64–2.81]	1.94 [1.48–2.40]	−0.28	−0.21
	CON	2.71 [1.70–3.71]	2.84 [1.78–3.91]	2.57 [1.75–3.39]	−0.27	−0.13
	*p*-value	0.589	0.558	0.339	0.476	0.336
	Cohen’s *d*	0.689	0.708	0.935		
Triglycerides (mmol/L) <1.70 mmol/L	VEG	0.58 [0.47–0.69]	0.70 [0.60–0.80]	0.67 [0.31–1.03]	−0.03	+0.09
	CON	0.80 [0.57–1.02]	1.15 [0.90–1.41]	1.95 [−0.16–4.05]	+0.79	+1.15
	*p*-value	0.188	**0.013**	0.457	0.360	0.114
	Cohen’s *d*	1.219	2.537	0.980		

VEG: vegan group (n = 9); CON: control group (n = 6). Group differences at each time point were assessed using 2-way ANOVA and a connected post hoc analysis. Group differences in temporal delta were calculated using an unpaired *t*-test. Differences between time points within the VEG and CON groups were calculated separately (2-way ANOVA) and are reported as *p*-values in each case (* *p* < 0.05). Effect sizes are reported as Cohen’s *d* for between-group comparisons. Data shown in bold indicate significant values.

**Table 5:** Biochemical parameters of the study groups at all measurement time points.

Parameter and Lab Reference Range	Group	t0 Mean [95% CI]	t1 Mean [95% CI]	t2 Mean [95% CI]	Δ(t2 − t1)	Δ(t2 − t0)
Hemoglobin (mmol/L) 8.34–10.7 mmol/L	VEG	8.98 [8.51–9.47]	8.96 [8.46–9.46]	9.13 [8.55–9.71]	+0.17	+0.15
	CON	9.07 [8.93–9.21]	9.23 [8.73–9.73]	9.22 [8.99–9.44]	+0.01	+0.15
	*p*-value	0.972	0.752	0.985	0.421	> 0.999
	Cohen’s *d*	0.174	0.458	0.143		
Ferritin (pmol/L) 33.7–674 pmol/L	VEG	155 [114–197]	143 [96.0–189]	133 [92.5–189]	−9.23	−22.0
	CON	220 [137–303]	183 [97.8–268]	177 [124–230]	−5.33	−42.9
	*p*-value	0.309	0.698	0.350	0.899	0.453
	Cohen’s *d*	1.005	0.579	0.842		
Magnesium (mmol/L) 0.73–1.06 mmol/L	VEG	0.88 [0.81–0.95]	0.95 [0.89–1.01]	0.88 [0.81–0.95]	−0.07	+0.00
	CON	0.90 [0.81–0.99]	1.00 [0.90–1.10]	0.85 [0.81–0.90]	−0.15	−0.05
	*p*-value	0.960	0.601	0.838	0.254	0.361
	Cohen’s *d*	0.232	0.658	0.353		
β–Carotene (μmol/L) 1.86–15.8 μmol/L	VEG	6.68 [3.13–10.3]	6.70 [4.07–9.32]	7.16 [3.54–10.8]	+0.46	+0.47
	CON	5.35 [3.32–7.38]	5.96 [3.49–8.43]	5.41 [3.63–7.19]	−0.55	+0.06
	*p*-value	0.838	0.950	0.698	0.257	0.619
	Cohen’s *d*	0.351	0.658	0.353		
Vitamin E (µmol/L) 17.6–53.6 µmol/L	VEG	24.6 [23.2–25.9]	23.4 [21.9–25.0]	23.2 [20.7–25.9]	−0.14	−1.25
	CON	29.7 [25.9–33.3]	29.5 [23.7–35.2]	31.3 [24.4–38.3]	+1.86	+1.74
	*p*-value	**0.044**	0.120	0.086	0.444	0.294
	Cohen’s *d*	1.962	1.615	1.650		
Folic acid (nmol/L) 7.02–46.4 nmol/L	VEG	18.4 [12.9–23.8]	19.9 [13.8–26.1]	23.6 [15.1–32.1]	+3.65	+5.23
	CON	21.2 [13.6–28.8]	23.5 [15.7–31.4]	20.2 [8.97–31.3]	−3.40	−1.07
	*p*-value	0.848	0.775	0.916	0.125	0.110
	Cohen’s *d*	0.271	0.618	0.834		
Zinc (μmol/L) 68.8–115 μmol/L	VEG	82.5 [76.8–88.1]	78.9 [72.7–85.0]	83.3 [76.8–89.8]	+4.44	+0.92
	CON	85.9 [75.2–96.6]	94.8 [70.1–120]	87.4 [76.5–98.4]	−7.34	+1.53
	*p*-value	0.868	0.413	0.821	0.114	0.836
	Cohen’s *d*	0.407	1.005	0.448		
Vitamin B_2_ (nmol/L) 364–983 nmol/L	VEG	501 [358–644]	526 [439–612]	518 [461–575]	−7.89	+16.8
	CON	553 [327–778]	579 [502–654]	580 [459–701]	+1.99	+27.4
	*p*–value	0.953	0.628	0.529	0.028	0.147
	Cohen’s *d*	0.271	0.618	0.834		
Vitamin A (μmol/L) 1.07–3.21 μmol /L	VEG	1.89 [1.71–2.06]	1.69 [1.38–2.00]	1.98 [1.82–2.15]	+0.29 *	+0.11
	CON	2.16 [1.80–2.52]	2.01 [1.65–2.38]	2.32 [2.01–2.64]	+0.32	+0.18
	*p*-value	0.330	0.332	0.120	0.913	0.595
	Cohen’s *d*	0.982	0.841	1.359		
Vitamin D (nmol/L) 75–250 nmol/L	VEG	106 [76.3–135]	112 [79.4–144]	110 [84.1–136]	−1.68	+4.17
	CON	110 [67.7–153]	111 [64.3–157]	92.4 [67.0–118]	−18.3	−17.9
	*p*-value	0.995	>0.999	0.599	0.108	**0.014**
	Cohen’s *d*	0.117	0.019	0.577		

VEG: vegan group (n = 9); CON: control group (n = 6). Group differences at each time point were assessed using 2-way ANOVA and a connected post hoc analysis. Group differences in temporal delta were calculated using an unpaired *t*-test. Differences between time points within the VEG and CON groups were calculated separately (2-way ANOVA) and are reported as *p*-values in each case (* *p* < 0.05). Effect sizes are reported as Cohen’s *d* for between-group comparisons. Data shown in bold indicate significant values.

**Table 6:** Anthropometric and exercise performance of the study groups.

Measure	Group	t0 Mean [95% CI]	t1 Mean [95% CI]	t2 Mean [95% CI]	Δ(t2 − t1)	Δ(t2 − t0)
Body weight (kg)	VEG	77.7 [71.0–84.3]	77.7 [71.2–84.3]	75.8 [69.7–81.8]	−1.94 **	−1.87
	CON	80.1 [72.2–88.0]	80.0 [72.3–87.6]	79.6 [71.7–87.6]	−0.37	−0.48
	*p*-value	0.923	0.932	0.741	**0.028**	0.120
	Cohen’s *d*	0.295	0.281	0.496		
FFM_b_ (kg)	VEG	69.1 [65.0–73.3]	69.3 [64.6–74.0]	67.3 [63.0–71.6]	−2.02 *	−1.85
	CON	68.9 [61.3–76.5]	69.2 [62.1–76.3]	68.3 [60.7–75.9]	−0.89	−0.59
	*p*-value	>0.999	>0.999	0.989	0.144	0.230
	Cohen’s *d*	0.037	0.017	0.153		
FFM_b_ (%)	VEG	89.5 [84.8–94.1]	89.5 [85.8–93.1]	89.1 [85.7–92.5]	−0.40	−0.42
	CON	86.0 [82.7–89.2]	86.4 [83.2–89.6]	85.8 [82.5–89.0]	−0.68	−0.22
	*p*-value	0.420	0.403	0.297	0.731	0.835
	Cohen’s *d*	0.685	0.723	0.836		
FM_b_ (%)	VEG	10.5 [5.87–15.2]	10.5 [6.89–14.2]	10.9 [7.54–14.4]	+0.40	+0.42
	CON	14.0 [10.8–17.3]	13.6 [10.4–16.8]	14.3 [11.0–17.5]	+0.68	+0.22
	*p*-value	0.420	0.403	0.297	0.731	0.835
	Cohen’s *d*	0.606	0.659	0.840		
FM_c_ (%)	VEG	11.4 [10.0–12.8]	11.4 [9.77–13.0]	10.8 [9.20–12.4]	−0.61	−0.62
	CON	13.0 [11.4–14.6]	12.9 [11.9–13.9]	12.6 [11.4–13.8]	−0.27	−0.38
	*p*-value	0.235	0.244	0.131	0.345	0.642
	Cohen’s *d*	0.948	0.849	1.043		
HR_rest_ (bpm)	VEG	81.6 [73.9–89.3]	63.3 [52.5–74.1]	66.1 [54.8–77.5]	+2.86	**−** 15.4 *
	CON	73.6 [57.0–90.2]	70.8 [60.1–81.5]	69.0 [50.5–87.5]	−1.80	−4.60
	*p*-value	0.628	0.543	0.981	0.380	0.080
	Cohen’s *d*	0.751	0.710	0.213		
HR_max_ (bpm)	VEG	189 [181–197]	183 ± [180–186]	185 [178–193]	+2.71	−3.86
	CON	188 [177–199]	183 [171–194]	181 [170–192]	−1.70	−7.00
	*p*-value	0.992	>0.999	0.779	0.248	0.591
	Cohen’s *d*	0.152	0.014	0.540		
Lac_IAT_ (mmol/L)	VEG	3.09 [2.65–3.52]	2.65 [2.24–3.07]	2.45 [2.12–2.79]	−0.19	−0.63
	CON	2.41 [2.00–2.83]	2.32 [1.75–2.90]	2.33 [2.01–2.66]	+0.01	−0.08
	*p*-value	**0.049**	0.598	0.885	0.292	0.071
	Cohen’s *d*	1.582	0.703	0.373		
Lac_max_ (mmol/L)	VEG	10.5 [9.34–11.7]	8.63 [5.85–11.4]	9.25 [6.73–11.8]	+0.61	−1.27
	CON	8.04 [6.25–9.83]	5.31 [3.60–7.02]	6.67 [3.66–9.69]	+1.37	−1.36
	*p*-value	**0.045**	0.089	0.315	0.590	0.957
	Cohen’s *d*	1.851	1.336	0.986		
v_IAT_ (km/h)	VEG	13.2 [12.5–18.9]	12.9 [12.1–13.6]	14.1 [13.5–14.7]	+1.21 *	+0.87 **
	CON	13.6 [12.7–14.6]	13.2 [12.1–14.3]	14.6 [13.3–15.9]	+1.42	+0.98 *
	*p*-value	0.720	0.900	0.726	0.728	0.726
	Cohen’s *d*	0.594	0.382	0.641		
v_max_ (km/h)	VEG	17.5 [16.6–18.4]	16.9 [15.1–17.3]	18.1 [17.4–18.8]	+1.88 *	+0.59
	CON	16.6 [15.9–17.4]	15.4 [14.2–16.7]	17.1 [15.8–18.3]	+1.67	+0.42
	*p*-value	0.247	0.572	0.275	0.784	0.810
	Cohen’s *d*	1.020	0.701	1.159		
Absolute VO_2max_ (mL/min)	VEG	4407 [3894–4920]	4226 [3731–4721]	4346 [3876–4816]	+120	−61.4
	CON	4542 [3980–5104]	4160 [3474–4846]	4176 [3615–4737]	+16.0	−366
	*p*-value	0.959	0.996	0.913	0.563	0.103
	Cohen’s *d*	0.261	0.121	0.349		

VEG: vegan group (ex. performance: n = 7; anthropometrics: n = 9); CON: control group (ex. performance: n = 5; anthropometrics: n = 6); FFM_b_/FM_b_: fat free mass/fat mass measured with BIA; FM_c_: fat mass measured with calipers; HR: heart rate; Lac: blood lactate concentration; VO_2_: oxygen capacity. Group differences at each time point were assessed using 2-way ANOVA and a connected post hoc analysis. Group differences in temporal delta were calculated using an unpaired *t*-test. Differences between time points within the VEG and CON groups were calculated separately (2-way ANOVA) and are reported as *p*-values in each case (* *p* < 0.05; ** *p* < 0.01). Effect sizes are reported as Cohen’s *d* for between-group comparisons. Data shown in bold indicate significant values.

## Data Availability

To protect the privacy of the participants, the data collected for this study cannot be shared publicly. However, the data are available upon reasonable request from the corresponding author.

## Supplementary Materials

The following supporting information can be downloaded at: https://www.mdpi.com/article/10.3390/nu17142351/s1, Table S1: Intake of energy, macro-, and micronutrients during the intervention (separate depiction of food and supplements).
