# Supplementary material for: The Effect of an 8-Week Vegan Diet on the Nutritional Status and Performance of Semi-Professional Soccer Players—Results of the VegInSoc Study
Source: Nutrients. 2025 Jul 17;17(14):2351. doi: 10.3390/nu17142351 (PMC12299004; doi:10.3390/nu17142351)
Supplement: Supplementary file 1 [file nutrients-17-02351-s001.zip › nutrients-3658457-supplementary.pdf]

nutrients

MDPI

Supplementary Material

The effect of an 8-week vegan diet on the nutritional status and performance of semi-professional soccer players – results of the VegInSoc study

Josefine Nebl [1,†], Pauline Bruns [2,†], Meike Meier [1], Frank Mayer [3], Martin Smollich [2,‡] and Markus Keller [1,*,‡]

1 Research Institute for Plant-Based Nutrition, 35444 Biebertal, Germany
2 Institute of Nutritional Medicine, University Hospital of Schleswig-Holstein, 23538 Lübeck, Germany
3 University Outpatient Clinic, Center of Sports Medicine, 14469 Potsdam, Germany
* Correspondence: keller@ifpe-giessen.de
† These authors contributed equally to this work.
‡ These authors contributed equally to this work.

Table S1. Intake of energy, macro-, and micronutrients during the intervention (separate depiction of food and supplements).

Measure	Group	Nutrient source	t0<br>mean [95% CI]	t1<br>mean [95% CI]	t2<br>mean [95% CI]
Energy (kcal)	VEG	Food	2463 [1870-2463]	2383 [1808-2958]	2609 [1980-3238]
		Supplement	24 [-9-58]	124 [22-225]	75 [-12-162]
	CON	Food	2545 [2019-3070]	2736 [1565-3908]	2565 [1311-3819]
		Supplement	39 [-28-105]	106 [-38-249]	41 [-30-112]
	p-value	Food	0.992	0.871	>0.999
	Cohen´s *d*	Food	0.121	0.432	0.050
	p-value	Supplement	0.949	0.992	0.853
	Cohen´s d	Supplement	0.299	0.142	0.345
Macronutrients					
Carbohydrates (En%)	VEG	Food	48.1 [44.1-52.1]	50.4 [46.2-54.6]	47.1 [40.6-53.5]
		Supplement	-0.15 [-0.55-0.25]	-1.18 [-2.12- -0.24]	-0.80 [-1.95-0.35]
	CON	Food	44.0 [35.0-53.0]	40.2 [32.5-48.0]	38.1 [34.3-42.0]
		Supplement	-0.61 [-1.65-0.42]	-0.71 [-1.76-0.34]	-0.53 [-1.42-0.37]
	p-value	Food	0.660	0.051	**0.045**
	Cohen´s *d*	Food	0.691	1.765	1.262
	p-value	Supplement	0.666	0.804	0.957
	Cohen´s *d*	Supplement	0.719	0.420	0.214
Fat (En%)	VEG	Food	32.4 [28.2-36.5]	33.1 [28.1-38.0]	36.2 [29.7-42.8]
		Supplement	-0.15 [-0.35-0.05]	0.65 [0.00-1.30]	0.35 [-0.19-0.89]
	CON	Food	35.4 [28.3-42.6]	37.3 [28.5-46.1]	40.4 [36.6-44.3]
		Supplement	-0.26 [-0.71-0.20]	-0.30 [-0.95-0.35]	-0.38 [-1.04-0.28]
	p-value	Food	0.730	0.660	0.506
	Cohen´s *d*	Food	0.561	0.635	0.0.586
	p-value	Supplement	0.932	0.070	0.150
	Cohen´s *d*	Supplement	0.350	1.262	1.117

Table S1 – continuing (1). Intake of energy, macro-, and micronutrients during the intervention (separate depiction of food and supplements).

Measure	Group	Nutrient source	t0<br>mean [95% CI]	t1<br>mean [95% CI]	t2<br>mean [95% CI]
EPA (mg)	VEG	Food	71.8 [11.7-132]	2.28 [0.30-4.26]	2.21 [0.54-3.88]
		Supplement	0.24 [-0.32-0.80]	27.8 [-36.3-91.8]	-0.42 [-1.18-0.34]
	CON	Food	390 [-245-1025]	153 [-14.8-321]	34.0 [13.7-54.3]
		Supplement	48.0 [-85.3-181]	32.0 [-56.9-121]	0 [0-0]
	p-value	Food	0.557	0.188	**0.035**
	Cohen´s *d*	Food	1.053	1.931	3.316
	p-value	Supplement	0.757	>0.999	0.557
	Cohen´s *d*	Supplement	0.771	0.053	0.521
DHA (mg)	VEG	Food	170 [28.8-310]	14.9 [3.89-25.8]	8.54 [-7.07-24.2]
		Supplement	0.21 [-0.27-0.68]	27.8 [-36.3-91.8	0.65 [-0.42-1.73]
	CON	Food	650 [-260-1560]	221 [-89.8-531]	40.8 [-1.79-83.4]
		Supplement	22.0 [-39.1-83.1]	14.7 [-26.1-55.4]	0 [0-0]
	p-value	Food	0.523	0.363	0.286
	Cohen´s *d*	Food	1.070	1.421	1.249
	p-value	Supplement	0.759	0.969	0.488
	Cohen´s *d*	Supplement	0.767	0.186	0.570
Protein (g/kg BW)	VEG	Food	1.23 [0.97-1.49]	0.89 [0.66-1.12]	1.04 [0.79-1.29]
		Supplement	0.04 [-0.02-0.10]	0.11 [0.03-0.19]	0.08 [-0.02-0.19]
	CON	Food	1.39 [1.13-1.65]	1.70 [0.78-2.62]	1.58 [0.74-2.42]
		Supplement	0.09 [-0.06-0.25]	0.15 [-0.06-0.36]	0.10 [-0.07-0.26]
	p-value	Food	0.643	0.195	0.396
	Cohen´s *d*	Food	0.690	1.614	1.052
	p-value	Supplement	0.803	0.894	0.996
	Cohen´s *d*	Supplement	0.578	0.331	0.102
Fiber (g)	VEG	Food	35.7 [21.8-51.5]	49.9 [36.2-65.9]	52.1 [38.6-68.1]
		Supplement	0.93 [-0.32-2.17]	1.20 [0.27-2.14]	1.29 [-0.34-2.92]
	CON	Food	28.0 [18.0-38.0]	32.3 [17.1-48.3]	25.5 [4.53-46.4]
		Supplement	0 [0-0]	0.31 [-0.29-0.92]	0 [0-0]
	p-value	Food	0.602	0.151	0.057
	Cohen´s *d*	Food	0.485	1.042	1.462
	p-value	Supplement	0.327	0.219	0.287
	Cohen´s *d*	Supplement	0.702	0.860	0.743
Vitamins					
A (RE, µg)	VEG	Food	1134 [457-1811]	1164 [415-1912]	1475 [750-220]
		Supplement	5.13 [-3.63-13.9]	3.27 [-1.63-8.17]	5.39 [-4.63-15.4]
	CON	Food	1243 [641-1845]	956 [406-1506]	1186 [857-1516]
		Supplement	1.68 [-1.18-4.54]	5.89 [-7.72-19.5]	1.82 [-1.32-4.96]
	p-value	Food	0.988	0.934	0.796
	Cohen´s *d*	Food	0.141	0.249	0.367
	p-value	Supplement	0.787	0.955	0.831
	Cohen´s *d*	Supplement	0.368	0.319	0.332
D (µg)	VEG	Food	2.11 [1.20-3.02]	3.45 [1.57-5.33]	1.59 [0.43-2.75]
		Supplement	10.2 [-11.0-31.4]	2.78 [-3.63-9.19]	12.1 [-9.46-33.6]
	CON	Food	3.81 [0.07-7.54]	3.04 [0.08-6.01]	14.1 [-17.7-45.8]
		Supplement	1.35 [-2.35-5.04]	13.3 [-12.2-38.9]	4.00 [-7.11-15.1]
	p-value	Food	0.633	0.988	0.709
	Cohen´s *d*	Food	0.852	0.167	0.841
	p-value	Supplement	0.745	0.691	0.829
	Cohen´s *d*	Supplement	0.393	0.772	0.344

Table S1 – continuing (2). Intake of energy, macro-, and micronutrients during the intervention (separate depiction of food and supplements).

Measure	Group	Nutrient source	t0<br>mean [95% CI]	t1<br>mean [95% CI]	t2<br>mean [95% CI]
E (mg)	VEG	Food	16.4 [11.7-21.1]	25.8 [18.8-32.7]	29.0 [19.8-38.2]
		Supplement	3.63 [-3.99-11.3]	4.16 [-4.51-12.8]	3.50 [-4.30-11.3]
	CON	Food	16.4 [10.2-22.6]	20.6 [8.05-33.1]	16.6 [3.45-29.7]
		Supplement	1.34 [-2.37-5.05]	4.15 [-6.99-15.3]	4.01 [-7.11-15.1]
	p-value	Food	>0.999	0.750	0.208
	Cohen´s *d*	Food	0.000	0.549	1.075
	p-value	Supplement	0.899	>0.999	>0.999
	Cohen´s *d*	Supplement	0.277	0.001	0.051
K (µg)	VEG	Food	153 [93.1-212]	168 [99.4-237]	212 [142-282]
		Supplement	4.57 [-2.69-11.8]	4.22 [-0.77-9.22]	5.94 [-2.66-14.6]
	CON	Food	175 [-24.3-375]	168 [25.4-310]	108 [13.3-203]
		Supplement	2.51 [-4.42-9.44]	21.2 [-16.6-58.9]	7.50 [-13.3-28.3]
	p-value	Food	0.989	>0.999	0.135
	Cohen´s *d*	Food	0.199	0.003	1.199
	p-value	Supplement	0.944	0.631	0.997
	Cohen´s *d*	Supplement	0.246	0.924	0.117
B_1 (mg)	VEG	Food	1.80 [1.22-2.37]	1.76 [1.22-2.30]	2.08 [1.55-2.60]
		Supplement	0.41 [-0.35-1.16	0.37 [-0.41-1.14]	0.37 [-0.40-1.14]
	CON	Food	1.49 [1.12-1.86]	2.44 [0.76-4.12]	1.87 [0.93-2.82]
		Supplement	0.27 [-0.40-0.93]	0.71 [-1.17-2.59]	0.71 [-1.19-2.61]
	p-value	Food	0.654	0.715	0.950
	Cohen´s *d*	Food	0.364	0.573	0.080
	p-value	Supplement	0.983	0.961	0.965
	Cohen´s *d*	Supplement	0.160	0.291	0.281
B_2 (mg)	VEG	Food	1.49 [1.08-1.90]	1.45 [0.94-1.96]	1.24 [0.83-1.66]
		Supplement	0.34 [-0.29-0.97]	0.33 [-0.32-0.98]	0.31 [-0.34-0.96]
	CON	Food	1.89 [1.22-2.57]	2.29 [0.86-3.72]	1.62 [0.83-2.41]
		Supplement	0.53 [-0.58-1.63]	1.08 [-1.50-3.66]	1.11 [-1.58-3.79]
	p-value	Food	0.522	0.461	0.652
	Cohen´s *d*	Food	0.748	0.986	0.660
	p-value	Supplement	0.975	0.856	0.849
	Cohen´s *d*	Supplement	0.222	0.542	0.558
Niacin (mg)	VEG	Food	34.7 [29.9-39.6]	29.5 [19.9-39.0]	24.3 [17.7-31.0]
		Supplement	0.49 [-0.27-1.26]	0.76 [0.16-1.36]	0.63 [-0.38-1.65]
	CON	Food	42.5 [31.3-53.6]	54.0 [22.7-85.2]	50.0 [20.9-79.1]
		Supplement	0.49 [-0.35-1.33]	1.06 [-0.60-2.73]	0.53 [-0.39-1.45]
	p-value	Food	0.360	0.260	0.193
	Cohen´s *d*	Food	1.057	1.383	1.680
	p-value	Supplement	>0.999	0.961	0.997
	Cohen´s *d*	Supplement	0.002	0.301	0.085
Pantothenic acid (mg)	VEG	Food	6.11 [4.25-7.96]	6.13 [3.09-9.18]	5.32 [3.09-9.18]
		Supplement	1.09 [-0.95-3.12]	0.95 [-1.10-3.00]	0.97 [-1.07-3.01]
	CON	Food	7.24 [3.47-11.0]	7.93 [1.97-13.9]	6.04 [2.59-9.49]
		Supplement	1.69 [-2.25-5.62]	4.06 [-6.41-14.5]	4.13 [-6.55-14.8]
	p-value	Food	0.872	0.874	0.960
	Cohen´s *d*	Food	0.430	0.421	0.251
	p-value	Supplement	0.980	0.845	0.846
	Cohen´s *d*	Supplement	0.211	0.583	0.583

Table S1 – continuing (3). Intake of energy, macro-, and micronutrients during the intervention (separate depiction of food and supplements).

Measure	Group	Nutrient source	t0 mean [95% CI]	t1 mean [95% CI]	t2 mean [95% CI]
B_6 (mg)	VEG	Food	2.46 [1.52-3.40]	1.96 [1.26-2.65]	2.23 [1.71-2.75]
		Supplement	0.07 [-0.03-0.17]	0.02 [0.00-0.04]	0.02 [-0.02-0.06]
	CON	Food	2.05 [1.48-2.62]	2.76 [1.17-4.35]	2.38 [0.88-3.88]
		Supplement	0.34 [-0.51-1.18]	0.90 [-1.49-3.30]	0.90 [-1.51-3.32]
	p-value	Food	0.779	0.592	0.993
	Cohen´s *d*	Food	0.390	0.769	0.170
	p-value	Supplement	0.817	0.745	0.746
	Cohen´s *d*	Supplement	0.658	0.789	0.787
Biotin (µg)	VEG	Food	65.7 [45.9-85.5]	69.6 [43.9-95.3]	70.1 [50.5-89.7]
		Supplement	0.66 [-0.35-1.67]	0.44 [-0.12-1.01]	0.64 [-0.44-1.73]
	CON	Food	72.7 [43.7-102]	51.8 [11.6-92.0]	47.9 [11.4-84.4]
		Supplement	9.58 [-10.8-29.9]	20.5 [-29.0-70.0	20.6 [-30.0-71.2]
	p-value	Food	0.945	0.735	0.482
	Cohen´s *d*	Food	0.278	0.536	0.827
	p-value	Supplement	0.644	0.691	0.705
	Cohen´s *d*	Supplement	0.936	0.870	0.849
Folic acid (µg)	VEG	Food	401 [262-540]	390 [278-503]	351 [221-481]
		Supplement	13.7 [-11.7-39.1]	11.0 [1.14-20.9]	12.1 [-8.69-33.0]
	CON	Food	399 [167-630]	334 [161-507]	235 [49.1-421]
		Supplement	14.5 [-24.0-52.9]	43.7 [-70.3-158]	41.3 [-71.4-154]
	p-value	Food	>0.999	0.872	0.517
	Cohen´s *d*	Food	0.012	0.390	0.715
	p-value	Supplement	>0.999	0.853	0.888
	Cohen´s *d*	Supplement	0.024	0.605	0.513
B_{12} (µg)	VEG	Food	3.66 [1.90-5.43]	0.44 [0.06-0.83]	0.65 [0.09-1.21]
		Supplement	19.9 [-23.5-63.3]	83.4 [-52.5-219]	83.4 [-52.6-219]
	CON	Food	5.76 [3.96-7.55]	6.66 [2.98-10.3]	6.15 [1.49-10.8]
		Supplement	1.04 [-1.47-3.54]	69.3 [-123-261]	2.66 [-4.34-9.66]
	p-value	Food	0.167	**0.027**	0.087
	Cohen´s *d*	Food	1.022	3.531	2.446
	p-value	Supplement	0.719	0.998	0.504
	Cohen´s *d*	Supplement	0.410	0.083	0.559
C (mg)	VEG	Food	178 [93.8-263]	225 [125-325]	157 [97.6-215]
		Supplement	35.0 [-41.5-112]	53.0 [-28.0-134]	42.3 [-33.9-118]
	CON	Food	149 [84.8-212]	116 [25.9-205]	86.6 [34.1-139]
		Supplement	213 [-379-806]	174 [-307-655]	240 [-426-908]
	p-value	Food	0.879	0.187	0.138
	Cohen´s *d*	Food	0.314	0.956	1.041
	p-value	Supplement	0.836	0.895	0.841
	Cohen´s *d*	Supplement	0.621	0.505	0.618
Minerals					
K (g)	VEG	Food	3.59 [2.59-4.80]	3.74 [2.44-4.93]	3.93 [2.82-5.05]
		Supplement	0.04 [-0.02-0.10]	0.28 [0.06-0.50]	0.12 [-0.05-0.29]
	CON	Food	3.47 [2.76-4.17]	3.43 [1.54-5.33]	3.00 [1.19-4.82]
		Supplement	0.18 [-0.13-0.50]	0.25 [-0.05-0.54]	0.20 [-0.15-0.54]
	p-value	Food	0.994	0.980	0.635
	Cohen´s *d*	Food	0.108	0.198	0.638
	p-value	Supplement	0.630	0.997	0.927
	Cohen´s *d*	Supplement	0.892	0.099	0.346

Table S1 – continuing (4). Intake of energy, macro-, and micronutrients during the intervention (separate depiction of food and supplements).

Measure	Group	Nutrient source	t0<br>mean [95% CI]	t1<br>mean [95% CI]	t2<br>mean [95% CI]
Ca (mg)	VEG	Food	960 [692-1228]	894 [659-1128]	851 [609-1092]
		Supplement	48.8 [-53-150]	70.5 [-40-181]	56.3 [-53-166]
	CON	Food	1198 [812-1585]	1267 [557-1976]	1161 [303-2020]
		Supplement	95.8 [-70-261]	118 [-76-313]	120 [-99-338]
	p-value	Food	0.527	0.543	0.769
	Cohen´s *d*	Food	0.707	0.903	0.654
	p-value	Supplement	0.905	0.930	0.885
	Cohen´s *d*	Supplement	0.354	0.324	0.409
Mg (mg)	VEG	Food	496 [359-633]	573 [428-866]	660 [452-866]
		Supplement	47.5 [-13-108]	173 [20-325]	81.7 [-22-186]
	CON	Food	484 [383-584]	504 [256-753]	435 [126-744]
		Supplement	61.5 [-82-205]	77.0 [-39-193]	50.6 [-63-164]
	p-value	Food	0.997	0.908	0.388
	Cohen´s *d*	Food	0.081	0.356	0.855
	p-value	Supplement	0.994	0.570	0.945
	Cohen´s *d*	Supplement	0.150	0.560	0.255
Fe (mg)	VEG	Food	17.5 [10.8-24.2]	20.4 [14.3-26.4]	20.6 [14.6-26.7]
		Supplement	0.90 [-0.29-2.10]	8.31 [-3.91-20.5]	6.88 [-5.68-19.4]
	CON	Food	15.8 [11.0-20.6]	20.3 [10.9-29.6]	16.7 [5.06-28.4]
		Supplement	0.09 [-0.06-0.24]	0.76 [-0.45-1.98]	0.10 [-0.07-0.26]
	p-value	Food	0.944	>0.999	0.839
	Cohen´s *d*	Food	0.232	0.013	0.463
	p-value	Supplement	0.398	0.475	0.575
	Cohen´s *d*	Supplement	0.641	0.581	0.508
Zn (mg)	VEG	Food	12.3 [9.48-15.1]	11.0 [8.15-13.9]	11.9 [9.21-14.6]
		Supplement	1.98 [-1.81-5.77]	2.01 [-1.90-5.93]	1.88 [-2.02-5.77]
	CON	Food	13.5 [9.78-17.2]	18.9 [8.42-29.4]	17.2 [6.64-27.7]
		Supplement	0.74 [-1.20-2.67]	2.23 [-3.59-8.04]	2.08 [-3.57-7.73]
	p-value	Food	0.886	0.286	0.569
	Cohen´s *d*	Food	0.355	1.364	0.928
	p-value	Supplement	0.875	>0.999	>0.999
	Cohen´s *d*	Supplement	0.302	0.043	0.041
I (μg)	VEG	Food	103 [60.1-146]	86.0 [61.1-111]	84.6 [47.1-122]
		Supplement	0.75 [-0.20-1.70]	0.45 [-0.06-0.95]	0.84 [-0.27-1.95]
	CON	Food	132 [45.9-218]	134 [44.2-223]	82.6 [30.3-125]
		Supplement	21.8 [-22.0-65.7]	40.8 [-54.0-136]	42.9 [-57.9-144]
	p-value	Food	0.831	0.527	>0.999
	Cohen´s *d*	Food	0.480	0.966	0.042
	p-value	Supplement	0.583	0.661	0.673
	Cohen´s *d*	Supplement	1.033	0.915	0.897

VEG: vegan group (n = 9), CON: control group (n = 5), RE: Retinol equivalents; dietary intake included supplement intake. Group differences at respective time points were assessed with a 2-way ANOVA and connected post-hoc analysis. Effect sizes were reported as Cohen's *d* for between-group comparisons.
